# Autistic and Attention Deficit/Hyperactivity Disorder Traits Are Associated with Suboptimal Performance among Japanese University Students

**DOI:** 10.31662/jmaj.2020-0001

**Published:** 2020-07-07

**Authors:** Tomoko Suzuki, Koji Wada, Basilua Andre Muzembo, Nlandu R Ngatu, Shiho Yoshii, Shunya Ikeda

**Affiliations:** 1 Department of Public Health, School of Medicine, International University of Health and Welfare (IUHW), Narita, Japan; 2 Graduate School of Medicine, International University of Health and Welfare (IUHW), Tokyo, Japan

**Keywords:** ADHD, ASD, attention deficit/hyperactivity disorder, autism spectrum disorder, autistic trait, class attendance, presenteeism, student performance

## Abstract

**Introduction:**

Recent estimates suggest that there is a substantial number of Japanese students with developmental disabilities. This study aimed to examine potential associations between autistic, autistic subcomponents, and attention deficit/hyperactivity disorder (ADHD) traits with student performance (as measured by presenteeism) and class attendance among Japanese university students.

**Methods:**

Participants comprised 721 students from different regions of Japan who completed a self-administered internet survey. Autistic and ADHD traits were measured using an abridged version of the autism spectrum quotient (AQ-Short) and adult ADHD self-report scale (ASRS). Presenteeism, which is an indicator of student performance, was assessed using the modified World Health Organization Health and Work Performance Questionnaire. Class attendance during the past year was self-reported by participants.

**Results:**

Students with high levels of autistic traits and high levels of ADHD traits were significantly more likely to report poor student performance (odds ratio [OR] = 3.07, 95% confidence interval [95% CI]: 1.90–4.96; and OR = 2.13, 95% CI: 1.32–3.42, respectively). Regarding autistic trait subcomponents, students with high levels of preference for routine (OR = 2.39, 95% CI: 1.38–4.13) and high levels of difficulties with social skills (OR = 1.81, 95% CI: 1.03–3.18) were also significantly more likely to report poor student performance. There were borderline significant associations between traits of attention-switching difficulties and poor student performance (OR = 1.78, 95% CI: 1.00–3.15). Regarding ADHD trait subcomponents, students with high levels of inattention (OR = 2.88, 95% CI: 1.32–6.26) were also significantly more likely to report poor student performance. Students with both high levels of autistic traits and high levels of ADHD traits were more likely to report poor student performance than those with high levels of only one trait type. There were, however, no statistically significant associations between these traits and low class attendance risk.

**Conclusions:**

Sickness presenteeism was significantly associated with high levels of both autistic traits and ADHD traits among Japanese university students.

## Introduction

Recent estimates suggest that the number of students diagnosed with or suspected of having developmental disabilities is substantial in Japan ^(1)^, and, no doubt, both clinically diagnosed and as-yet undiagnosed (so-called typical) students will require additional support. At present, in Japan, almost all (97.1%) the developmental disabilities among university students comprise autism spectrum disorder (ASD) and attention deficit/hyperactivity disorder (ADHD) ^(1)^. Both these neurodevelopmental disorders are well known to cause severe impairment and morbidity ^(2)^. On the other hand, it is well known that the early detection of mental health problems enables early intervention and support, which may improve health and prevent further mental deterioration ^(3)^.

Auditory filtering difficulties, sensory under-responsiveness, and sensory seeking are associated with academic underachievement among students with ASD in mainstream classrooms ^(4)^, while students with ASD will exhibit high levels of behavioral and emotional difficulties at school, including attention difficulties. One study reported high rates of academic underachievement in students with ASD, with an intelligence quotient (IQ) within the average range (54% of students with ASD vs. 8% of typically developing students) ^(5)^. We hypothesized that students with ASD, therefore, would exhibit poor student performance owing to difficulties caused by their ASD symptoms and would subsequently show poor academic achievement. A sociodemographic gradient may also exist, as some research suggests that ASDs are more prevalent among less-educated adults ^(6)^, and that autistic traits are generally more prevalent among those of lower socioeconomic status (SES) ^(7)^. These findings suggest that autistic traits are probably concentrated among low SES groups because individuals with high autistic traits will exhibit poor student performance as a result of difficulties caused by their ASD symptoms.

ADHD symptoms have many negative life impacts and university students with these issues are known to have a lower quality of life than their peers without ADHD ^(8)^. For example, it has been shown that university students with ADHD symptoms tend to exhibit low academic achievement, as they find it difficult to concentrate on their studies and complete assignments ^(9), (10)^. Furthermore, a previous study of workplace costs found that ADHD was associated with a 4–5% reduction in work performance and the relative odds of sickness absence were doubled ^(11)^. Adult ADHD is a common disorder in the US civilian labor force and is associated with substantial losses in work time ^(12)^. These findings suggest that individuals with high levels of ADHD traits will exhibit poor student performance owing to difficulties caused by ADHD symptoms, not because of low ability.

Presenteeism is an indicator of student performance. Presenteeism is a tendency for individuals to go to work despite suboptimal functioning because of illness or other medical conditions ^(13)^. Initial reports of presenteeism were based on studies of workers; however, subsequent reports indicate that presenteeism also occurs among students ^(14), (15)^. Some research has also demonstrated a relationship between poor work performance (sickness presenteeism) and sickness absence ^(16), (17), (18)^, including one study from Japan where poor work performance (more sickness presenteeism) was related to higher absence rates due to mental health issues ^(19)^. This suggests that poor student performance may lead to low class attendance, and therefore, that individuals with high levels of autistic and ADHD traits are more likely to demonstrate lower class attendance rates.

Although research has examined some associations between student performance and developmental disability traits (autistic traits and ADHD traits) with typical intelligence ^(5), (9)^, the simultaneous evaluation of both autistic traits and ADHD traits is rare. In addition, most studies on developmental disability traits and poor student performance have utilized academic achievement as an indicator of student performance ^(5), (9)^. When excluding the effect of ability, academic achievement tends to be affected by student performance factors, such as presenteeism. Gender differences have also been shown to be important considerations within Japanese academic settings ^(20)^. As such, the current study examined potential associations between autistic traits, autistic trait subcomponents, and ADHD traits with poor student performance (sickness presenteeism), and class attendance rates among Japanese university students. Findings from the current study will provide useful data to help devise appropriate student support services and policies.

## Materials and Methods

### Participants

Participants comprised Japanese university students who completed a self-administered internet-based questionnaire survey in December 2016. The sample was drawn from a pool of individuals who had previously registered with a Japanese online survey company (Macromill Inc., Tokyo, Japan), comprising approximately 1.2 million registered members across all Japanese prefectures. The survey company guarantees the most recent internet user representation, and groups are not biased toward specific attributes. A total of 128,807 registered university students were selected randomly and invited by email to participate in the study. In the first email, screening was conducted to confirm that the respondent was a university student. In the second email, a greeting and a questionnaire were sent to respondents who had confirmed that they were university students in the screening. The greeting explained that the study theme was developmental disability traits among university students. The survey was closed when 1,030 students had completed the questionnaire. The company provides 103% of the desired sample size. The company was asked to provide 1,000 samples, and the ratio of each course was 1:6:1:2 for junior college (2-year university course), 4-year university course, 6-year university course, and graduate students. At the close of the survey, screening emails had been sent to 73,603 registered students, and questionnaire emails sent to 2,314 respondents who answered the screening. The only inclusion criterion was that participants had to be currently enrolled in either a 4- or a 6-year university course. When junior college students and graduate students were excluded from the total of 1,030, the final analysis target was 721. The questionnaire was configured to finish automatically when all responses had been completed.

A total of 721 students aged 18–30 years completed the survey. The sociodemographic characteristics assessed were age, gender, length of study (4- or 6-year university course), faculty, and leisure-time physical activity (no or yes). Other characteristics assessed were skipping breakfast (yes or no) and living arrangements (alone or not alone). The students’ field of study was classified into four groups: science and engineering (science, engineering, and science and technology), medicine (medicine, pharmaceutical sciences, nursing, health sciences, and home economics), literature (letters, law, economics, and sociology), and others.

### Autistic traits

The abridged version of the autism spectrum quotient (AQ-Short) is a self-administered test that measures the degree to which an adult with typical intelligence has traits associated with ASD ^(21)^. It is based on the AQ questionnaire created by Baron-Cohen et al. ^(22)^ and is used worldwide. AQ-Short total scores have been shown to differentiate accurately between participants and individuals clinically diagnosed with Asperger’s syndrome. The distribution of the AQ-Short scores is approximately normal across the Dutch general population and English student population ^(21)^.

AQ-Short subcomponent scores were used as a measure of specific autistic traits and comprise two higher-order subcomponents assessing a fascination for numbers and patterns (Numbers/Patterns, 5 items) and social behavioral difficulties (Social behavior, 23 items) ^(21)^. The social behavior subcomponent further comprises four lower-order subcomponents assessing difficulties with imagination (Imagination, 8 items), a preference for routine (Routine, 4 items), difficulties with social skills (Social skills, 7 items), and attention-switching difficulties (Switching, 4 items) ^(21)^. Participants respond to statements using a 4-point Likert scale: 1 = definitely agree; 2 = slightly agree; 3 = slightly disagree; and 4 = definitely disagree. Scoring is reversed for items on which an “agree” response reflects the presence of a characteristic of autism. Scores are summed; a minimum AQ-Short score of 28 indicates no autistic traits and a maximum score of 112 indicates a full autistic phenotype. The accuracy of the AQ-Short in differentiating individuals with ASD from controls was evaluated using receiver operating characteristic curve analysis. The area under the curve (AUC) was 0.97. The cutoff was ≥ 70, and the sensitivity and specificity were 0.94 and 0.91, respectively ^(21)^. We used a Japanese version of the AQ-Short form ^(7)^, with all 28 items needing to be answered for the AQ-Short data to be considered acceptable.

The AQ-Short was used instead of the original AQ for the following reasons: first, the correlations between the AQ and AQ-Short were very high and significant in all samples (Control sample: r = 0.94, *p* < 0.001; ASD sample: r = 0.95, *p* < 0.001) ^(21)^. Second, participants are burdened with 50 questions in the AQ questionnaire, compared with 28 questions in the AQ-Short questionnaire. Third, there are several short versions of the AQ. However, the AQ-Short contains a subcomponent to measure the autistic phenotype “a fascination for numbers and patterns,” which was uniquely identified in our previous study as a positive aspect on which workers of greater SES scored highly ^(7)^.

### ADHD traits

The adult ADHD self-report scale (ASRS), which was developed in conjunction with the World Health Organization (WHO) and the Workgroup on Adult ADHD ^(23)^, was administered to determine participants’ ADHD traits. The ASRS Symptom Checklist comprises 18 DSM-IV adult ADHD criteria ^(24)^, while the ASRS Screener (a short form of the ASRS) comprises 6 of the 18 ASRS questions found to be most predictive of symptoms consistent with ADHD. The 6 questions represent the 4 symptoms of inattention and 2 symptoms of impulsivity/hyperactivity that characterize ADHD ^(23)^. The ASRS Screener items assessing inattention (Inattention, 4 items) and impulsivity/hyperactivity (Impulsivity/Hyperactivity, 2 items) were defined as ASRS subcomponents in this study. Each question asks how often a particular symptom has occurred over the past 6 months, with responses rated on a 0–4 scale: never (0), rarely (1), sometimes (2), often (3), and very often (4). Higher scores indicate a higher level of ADHD traits. We utilized a Japanese version of the ASRS for this study ^(25)^, and all 6 items had to be answered for the ASRS data to be considered complete. Data obtained using the DSM-IV ASRS screening scale can also be used to determine ADHD traits according to the DSM-5 criteria ^(2)^, by applying scoring rules developed to update the DSM-IV ASRS screening scale to the DSM-5 version of the screening scale ^(26), (27)^. The DSM-IV ASRS scale can therefore be used as a practical screening tool for the DSM-5 adult ADHD, despite several of the screening scale questions not necessarily reflecting DSM-5 symptoms (at the optimal threshold (cutoff of ≥ 11): sensitivity, 84.2%; specificity, 89.5%; AUC, 0.87) ^(26)^. This results in a minimum score of 0 and a maximum score of 20.

### Student performance

Student performance was assessed using the modified WHO Health and Work Performance Questionnaire (WHO-HPQ) ^(28), (29), (30)^, a self-reporting instrument designed to estimate the workplace costs of health problems in terms of self-reported reduced job performance (sickness presenteeism). Illness in the workplace can result in lost productivity, while “presenteeism” refers to productivity decreases among employees who are present but functioning at suboptimal capacity owing to illness or other medical conditions ^(28), (29)^. Although there are various definitions of the term “presenteeism,” all pertain to being physically present at work. There is also a Japanese version of the HPQ short form ^(19), (31)^.

We utilized a version of the HPQ short form modified for its suitability among student populations. Presenteeism can be calculated as the difference between actual and potential performance of self over the past 28 days ^(30)^. The modified HPQ measures presenteeism using the following question: “On a scale from 0 to 10, where 0 is the worst student performance and 10 is the best student performance, how would you rate your overall studying performance on the days you studied during the past four weeks?” A presenteeism score is obtained by multiplying the respondent’s response to the question by 10. Presenteeism has a lower bound of 0 (total lack of performance during time on the job) and an upper bound of 100 (no lack of performance during time on the job). A low presenteeism score indicates a low level of performance. In this study, poor student performance was defined as having a presenteeism score ≤ 40 ^(32)^.

### Class attendance

The percentage of attendance was measured using a self-administered questionnaire comprising the following question:****“Of the number of days that you were expected to attend during the past year, what percentage of days did you attend?” The number of class hours in Japanese universities varies greatly according to the field of study. For this reason, in this study, the group with the lowest quartile defined as “low attendance” had a percentage of attendance of below 80%.

### Statistical Analysis

Categorical variables were presented as percentages. AQ-Short scores, AQ-Short subcomponent scores, and ASRS scores were used to indicate the trait of developmental disability. For categorical variables, the chi-squared -test was used to compare subjects with poor student performance or low class attendance to those without poor student performance or low class attendance. Spearman correlation analysis was used to evaluate the relationship between the score quartiles of developmental disability traits, poor student performance category score, and low attendance category score, and to identify the collinearity between the score quartiles of developmental disability traits. Ordinal numbers 1–4 were assigned quartile categories of each developmental disability trait. Logistic regression analysis was undertaken to estimate the odds ratio (OR) and 95% confidence interval (95% CI) for poor student performance and low attendance, using the score quartiles of developmental disability traits. Only the ADHD trait subcomponent “Hyperactivity–Impulsivity” was analyzed in tertiles because it could not be divided into quartiles from the distribution. Goodness of fit of the logistic model was assessed using the Hosmer–Lemeshow test.

Univariate analysis was first performed, with multivariate models then adjusted for age, gender, length of study (4- or 6-year university course), field of study (science and engineering, medicine, literature, and others), leisure-time physical activity (no vs. yes), skipping breakfast (yes vs. no), and living arrangement (alone vs. not alone). Autistic traits, autistic trait subcomponents, ADHD traits, and ADHD trait subcomponents as the traits of developmental disability comprised the exposure variables of interest. In the first model, autistic traits and ADHD traits were entered simultaneously. In the second model, the five autistic trait subcomponents and ADHD traits were entered simultaneously, and stepwise multivariate logistic regression was conducted. In the third model, autistic traits and ADHD trait subcomponents were entered simultaneously. To examine combinations when scores on one or both autistic traits and ADHD traits were high, logistic regression analysis was used to estimate the OR and 95% CI for poor student performance and low attendance, using the “high” score and “low” score on the developmental disability traits. The 4th quartile (highest) of the developmental disability traits was defined as “high” and the 1st quartile (lowest) to the 3rd quartile of these was defined as “low.” Two-tailed *p*-values of < 0.05 were considered statistically significant. All analyses were conducted using SPSS (version 24 for Windows, IBM Inc., New York, USA).

### Ethics statement

Ethical approval was obtained from the International University of Health and Welfare Ethics Committee (No. 16-Ig-62) and the Kitasato University School of Medicine Ethics Committee (No. B16-160). The study was conducted in accordance with the standards specified in the 1964 Declaration of Helsinki. Participant responses implied voluntary consent for participation.

## Results

Participant demographics are displayed in Table 1. A total of 721 students (218 men and 503 women) participated in this study, with the most frequent course duration being 4 years in length (85.7%). The most common field of study was literature (37.7%). Regarding lifestyle factors, around two-thirds of the students reported no leisure-time physical activity, approximately a quarter were skipping breakfast, and about one-third lived alone.

**Table 1.** Participant Characteristics (*n* = 721).

	*n *(%)
**Age range (years)**	
18–19	136 (18.9)
20–22	487 (67.5)
23–30	98 (13.6)
**Male gender**	218 (30.2)
**Course duration**	
6-year university course	103 (14.3)
4-year university course	618 (85.7)
**Field of study**	
Science and engineering	83 (11.5)
Medicine	170 (23.6)
Literature	272 (37.7)
Others	196 (27.2)
**No leisure-time physical activity**	446 (61.9)
**Skipping breakfast**	182 (25.2)
**Living alone**	251 (34.8)

Supplementary Table 1 shows participant scores on autistic traits and ADHD traits. Regarding autistic traits, the mean total, male, and female scores were approximately 70, and about half the participants scored above the cutoff value. Regarding ADHD traits, the mean total, male, and female scores were approximately 8, and the percentage of participants above the cutoff value was 18.4%, 20.6%, and 17.5%, respectively. The percentage of the total sample, males, and females above the cutoff value for both autistic traits and ADHD traits was 11.2%, 14.2%, and 9.9%, respectively.

Table 2 shows Spearman correlations between autistic traits, autistic trait subcomponents, ADHD traits, poor student performance, and low attendance category score. Poor student performance score (≤40) was significantly positively associated with values for low attendance (< 80%), total and subcomponent autistic trait quartile scores, and ADHD trait quartile scores, except for quartile scores on the subcomponent autistic trait Numbers/Patterns. There were no significant associations between low attendance (< 80%) and total and subcomponent autistic trait quartile scores and ADHD trait quartile scores. The correlation results suggested that there would be no problem with multicollinearity in the subsequent regression analyses.

**Table 2.** Spearman Correlations between Autistic Traits, Autistic Trait Subcomponents, ADHD Traits, Poor Student Performance, and Low Attendance Category Score (*n* = 721).

	1	2	3	4	5	6	7	8	9
1. Poor student performance (presenteeism, ≤40)	1	0.16***	0.22***	-0.08*	0.16***	0.20***	0.19***	0.20***	0.16***
2. Low attendance (< 80%)		1	0.02	0.01	-0.01	0.03	0.01	0.03	0.07
***(Autistic traits)***									
3. AQ Short (quartiles)			1	0.05	0.59***	0.53***	0.71***	0.49***	0.15***
***(Autistic trait subcomponents)***									
4. Numbers/patterns (quartiles)				1	-0.18***	-0.14***	-0.20***	-0.29***	0.06
5. Imagination (quartiles)					1	0.25***	0.32***	0.32***	0.11**
6. Routine (quartiles)						1	0.35***	0.37***	0.14***
7. Social skills (quartiles)							1	0.34***	0.05
8. Switching (quartiles)								1	0.14***
***(ADHD traits)***									
9. ASRS (quartiles)									1

ADHD: attention deficit/hyperactivity disorder; AQ-Short: abridged version of the Autism Spectrum Quotient; ASRS: Adult ADHD Self-Report Scale. Based on the Spearman correlation analysis, ordinal numbers 1–4 were assigned quartile categories of each autistic trait, autistic trait subcomponents, and ADHD traits; *p < 0.05, **p < 0.01, ***p < 0.001.

Table 3 shows participant characteristics dichotomized according to the presence or absence of poor student performance and low class attendance. Compared with participants who did not report poor student performance, those with poor student performance were likely to be younger, to not engage in leisure-time physical activity, and more likely to skip breakfast. Compared with participants without low attendance, students reporting low attendance were more commonly enrolled in the literature faculty, less likely to be in the science and technology or medicine faculties, and more likely to skip breakfast.

**Table 3.** Participant Characteristics According to the Presence/Absence of Poor Student Performance (Presenteeism) and Low Class Attendance (*n *= 721).

	**Poor Performance**					**Low Attendance**				
	**Yes (presenteeism** **scores ≤40)**		**No (presenteeism** **scores >40)**		***p***	**Yes (class attendance <80%)**		**No (class attendance ≥80%)**		***p***
	*n*	(%)	*n*	(%)		*n*	(%)	*n*	(%)	
**Age range (years)**										
18–19	50	(21.6)	86	(17.6)	0.375	18	(14.8)	118	(19.7)	0.255
20–22	154	(66.4)	333	(68.1)		83	(68.0)	404	(67.4)	
23–30	28	(12.1)	70	(14.3)		21	(17.2)	77	(12.9)	
**Male gender**	66	(28.4)	152	(31.1)	0.472	44	(36.1)	174	(29.0)	0.124
**Course duration**										
6-year university course	29	(12.5)	74	(15.1)	0.345	12	(9.8)	91	(15.2)	0.123
4-year university course	203	(87.5)	415	(84.9)		110	(90.2)	508	(84.8)	
**Field of study**										
Science and engineering	26	(11.2)	57	(11.7)	0.808	9	(7.4)	74	(12.4)	0.001
Medicine	57	(24.6)	113	(23.1)		18	(14.8)	152	(25.4)	
Literature	91	(39.2)	181	(37.0)		64	(52.5)	208	(34.7)	
Others	58	(25.0)	138	(28.2)		31	(25.4)	165	(27.5)	
**No leisure-time physical activity**	159	(68.5)	287	(58.7)	0.011	76	(62.3)	370	(61.8)	0.913
**Skipping breakfast**	68	(29.3)	114	(23.3)	0.083	47	(38.5)	135	(22.5)	<0.001
**Living alone**	79	(34.1)	172	(35.2)	0.768	45	(36.9)	206	(34.4)	0.598

For categorical variables, the chi-squared test was used to compare students with poor performance or low class attendance with students without poor performance or low class attendance.

Table 4-1 and Supplementary Table 2 describe statistical associations between poor student performance (presenteeism) in university and scores on autistic traits, autistic trait subcomponents, and ADHD traits. Univariate analyses revealed that participants in the highest autistic trait score quartile and the highest ADHD trait score quartile were significantly more likely to report poor student performance: OR = 3.48 (95% CI: 2.19–5.53) and OR = 2.48 (95% CI: 1.57–3.91), respectively. In the first multivariate adjustment model (into which autistic traits and ADHD traits were entered simultaneously), the results were essentially unchanged. Participants in the highest autistic trait score quartile and the highest ADHD trait score quartile were significantly more likely to report poor student performance (OR = 3.07, 95% CI: 1.90–4.96; and OR = 2.13, 95% CI: 1.32–3.42, respectively). In the second multivariate adjustment model (into which five autistic trait subcomponents and ADHD traits had been entered simultaneously), participants in the highest score quartile for all subcomponents except two (Numbers/Patterns and Imagination) were more likely to report poor student performance. There was a significant difference for Routine traits (OR = 2.39, 95% CI: 1.38–4.13), Social skills traits (OR = 1.81, 95% CI: 1.03–3.18), and ADHD traits (OR = 2.24, 95% CI: 1.35–3.69). There were borderline statistically significant associations between the Switching traits and poor student performance (OR = 1.78, 95% CI: 1.00–3.15). There were no statistically significant associations between the Numbers/Patterns and the Imagination traits and poor student performance. The stepwise multivariate logistic regression results for the second multivariate adjustment model showed a significant difference for Routine traits (OR = 2.41, 95% CI: 1.42–4.11), Social skills traits (OR = 2.05, 95% CI: 1.20–3.51), Switching traits (OR = 2.01, 95% CI: 1.18–3.45), and ADHD traits (OR = 2.30, 95% CI: 1.41–3.73) (Supplementary Table 3). Furthermore, there were no statistically significant associations between autistic traits, autistic trait subcomponents, or ADHD traits and low class attendance in either the univariate model or the two multivariate models in the current study (Table 5-1, Supplementary Tables 4 and 5).

**Table 4-1.** Association between Poor Student Performance (Presenteeism) in University and Scores on Autistic Traits, Autistic Trait Subcomponents, and ADHD Traits (*n* = 721).

		Range of scores	No. of participants	No. of poor performance	**Univariate**		**Multivariate^a^**		**Multivariate^b^**	
					**OR (95% CI)**	***p***	**OR (95% CI)**	***p***	**OR (95% CI)**	***p***
**Autistic traits**										
AQ-Short	1^st^ Quartile (lowest)	40–66	197	44	1.00	-	1.00	-		
	2^nd^ Quartile	67–70	182	43	1.08 (0.67–1.74)	0.765	1.03 (0.63–1.68)	0.915		
	3^rd^ Quartile	71–75	192	70	2.00 (1.28–3.12)	0.002	1.92 (1.21–3.04)	0.006		
	4^th^ Quartile (highest)	76–104	150	75	3.48 (2.19–5.53)	<0.001	3.07 (1.90–4.96)	<0.001		
**Autistic trait subcomponents**										
Numbers/patterns	1^st^ Quartile (lowest)	5–9	192	75	1.00	-			1.00	-
	2^nd^ Quartile	10–11	192	61	0.73 (0.48–1.11)	0.136			0.90 (0.56–1.46)	0.675
	3^rd^ Quartile	12–13	166	44	0.56 (0.36–0.88)	0.012			0.76 (0.45–1.26)	0.285
	4^th^ Quartile (highest)	14–20	171	52	0.68 (0.44–1.05)	0.085			1.01 (0.60–1.70)	0.968
Imagination	1^st^ Quartile (lowest)	8–18	236	60	1.00	-			1.00	-
	2^nd^ Quartile	19–20	198	57	1.19 (0.78–1.81)	0.432			1.16 (0.73–1.84)	0.523
	3^rd^ Quartile	21–22	159	51	1.39 (0.89–2.16)	0.150			1.11 (0.68–1.81)	0.670
	4^th^ Quartile (highest)	23–32	128	64	2.93 (1.86–4.62)	<0.001			1.51 (0.88–2.59)	0.132
Routine	1^st^ Quartile (lowest)	4–9	169	31	1.00	-			1.00	-
	2^nd^ Quartile	10–10	173	57	2.19 (1.32–3.62)	0.002			2.03 (1.19–3.48)	0.010
	3^rd^ Quartile	11–11	164	43	1.58 (0.94–2.67)	0.085			1.37 (0.78–2.40)	0.280
	4^th^ Quartile (highest)	12–16	215	101	3.94 (2.46–6.33)	<0.001			2.39 (1.38–4.13)	0.002
Social skills	1^st^ Quartile (lowest)	7–15	186	48	1.00	-			1.00	-
	2^nd^ Quartile	16–18	225	53	0.89 (0.57–1.39)	0.598			0.78 (0.48–1.27)	0.321
	3^rd^ Quartile	19–21	175	59	1.46 (0.93–2.3)	0.101			1.18 (0.71–1.97)	0.515
	4^th^ Quartile (highest)	22–28	135	72	3.29 (2.05–5.26)	<0.001			1.81 (1.03–3.18)	0.040
Switching	1^st^ Quartile (lowest)	6–9	214	51	1.00	-			1.00	-
	2^nd^ Quartile	10–11	274	77	1.25 (0.83–1.88)	0.288			1.03 (0.66–1.62)	0.893
	3^rd^ Quartile	12–12	111	37	1.60 (0.97–2.65)	0.069			0.99 (0.55–1.78)	0.969
	4^th^ Quartile (highest)	13–16	122	67	3.89 (2.42–6.26)	<0.001			1.78 (1.00–3.15)	0.050
**ADHD traits**										
ASRS	1^st^ Quartile (lowest)	0–7	174	43	1.00	-	1.00	-	1.00	-
	2^nd^ Quartile	8–8	141	36	1.05 (0.63–1.74)	0.868	1.02 (0.60–1.73)	0.952	1.23 (0.71–2.15)	0.459
	3^rd^ Quartile	9–9	232	75	1.46 (0.94–2.26)	0.095	1.22 (0.77–1.93)	0.408	1.35 (0.83–2.20)	0.220
	4^th^ Quartile (highest)	10–20	174	78	2.48 (1.57–3.91)	<0.001	2.13 (1.32–3.42)	0.002	2.24 (1.35–3.69)	0.002

Poor performance: presenteeism scores ≤ 40; OR: odds ratio; CI: confidence interval; AQ-Short: abridged version of the Autism Spectrum Quotient; ASRS: Adult ADHD Self-Report Scale; ADHD: attention deficit/hyperactivity disorder. Logistic regression was performed to estimate the OR and 95% CI for poor performance, using the score quartiles of the trait of developmental disability. The models were adjusted for age, gender, length of study, field of study, leisure-time physical activity, skipping breakfast, and living arrangement. ^a^In the first model, autistic traits and ADHD traits were entered simultaneously. ^b^In the second model, five autistic trait subcomponents and ADHD traits were entered simultaneously. The Hosmer–Lemeshow test showed a chi-square value of 5.44 and a *p*-value of 0.71 in the first model, and a chi-square value of 7.54 and a *p*-value of 0.48 in the second model.

**Table 5-1.** Association between Low Class Attendance in University and Scores on Autistic Traits, Autistic Trait Subcomponents, and ADHD Traits (*n* = 721).

		Range of scores	No. of participants	No. of low class attendance	Univariate		Multivariate^a^		Multivariate^b^	
					**OR (95% CI)**	***p***	**OR (95% CI)**	***p***	**OR (95% CI)**	***p***
**Autistic traits**										
AQ-Short	1^st^ Quartile (lowest)	40–66	197	34	1.00	-	1.00	-		
	2^nd^ Quartile	67–70	182	27	0.84 (0.48–1.45)	0.522	0.84 (0.47–1.48)	0.536		
	3^rd^ Quartile	71–75	192	33	1.00 (0.59–1.68)	0.985	0.92 (0.53–1.60)	0.768		
	4^th^ Quartile (highest)	76–104	150	28	1.10 (0.63–1.91)	0.735	1.00 (0.56–1.79)	0.996		
**Autistic trait subcomponents**										
Numbers/patterns	1^st^ Quartile (lowest)	5–9	192	32	1.00	-			1.00	-
	2^nd^ Quartile	10–11	192	29	0.89 (0.51–1.54)	0.675			1.05 (0.57–1.91)	0.883
	3^rd^ Quartile	12–13	166	33	1.24 (0.72–2.12)	0.432			1.68 (0.92–3.07)	0.094
	4^th^ Quartile (highest)	14–20	171	28	0.98 (0.56–1.71)	0.940			1.09 (0.57–2.08)	0.802
Imagination	1^st^ Quartile (lowest)	8–18	236	41	1.00	-			1.00	-
	2^nd^ Quartile	19–20	198	34	0.99 (0.60–1.63)	0.956			0.92 (0.53–1.58)	0.759
	3^rd^ Quartile	21–22	159	25	0.89 (0.52–1.53)	0.667			0.74 (0.41–1.35)	0.325
	4^th^ Quartile (highest)	23–32	128	22	0.99 (0.56–1.74)	0.964			0.86 (0.44–1.69)	0.661
Routine	1^st^ Quartile (lowest)	4–9	169	26	1.00	-			1.00	-
	2^nd^ Quartile	10–10	173	27	1.02 (0.57–1.83)	0.955			1.03 (0.55–1.93)	0.935
	3^rd^ Quartile	11–11	164	30	1.23 (0.69–2.19)	0.479			1.35 (0.72–2.54)	0.347
	4^th^ Quartile (highest)	12–16	215	39	1.22 (0.71–2.10)	0.475			1.17 (0.61–2.24)	0.635
Social skills	1^st^ Quartile (lowest)	7–15	186	37	1.00	-			1.00	-
	2^nd^ Quartile	16–18	225	29	0.60 (0.35–1.01)	0.056			0.54 (0.31–0.96)	0.036
	3^rd^ Quartile	19–21	175	27	0.74 (0.43–1.27)	0.268			0.64 (0.35–1.17)	0.147
	4^th^ Quartile (highest)	22–28	135	29	1.10 (0.64–1.90)	0.728			0.91 (0.47–1.78)	0.780
Switching	1^st^ Quartile (lowest)	6–9	214	36	1.00	-			1.00	-
	2^nd^ Quartile	10–11	274	43	0.92 (0.57–1.49)	0.737			0.89 (0.52–1.51)	0.659
	3^rd^ Quartile	12–12	111	13	0.66 (0.33–1.30)	0.224			0.75 (0.35–1.61)	0.459
	4^th^ Quartile (highest)	13–16	122	30	1.61 (0.93–2.78)	0.086			1.49 (0.76–2.92)	0.244
**ADHD traits**										
ASRS	1^st^ Quartile (lowest)	0–7	174	26	1.00	-	1.00	-	1.00	-
	2^nd^ Quartile	8–8	141	16	0.73 (0.37–1.42)	0.352	0.76 (0.39–1.51)	0.439	0.82 (0.41–1.67)	0.586
	3^rd^ Quartile	9–9	232	45	1.37 (0.81–2.32)	0.243	1.43 (0.82–2.48)	0.207	1.54 (0.87–2.73)	0.140
	4^th^ Quartile (highest)	10–20	174	35	1.43 (0.82–2.50)	0.206	1.45 (0.81–2.59)	0.209	1.45 (0.79–2.64)	0.227

Low attendance: class attendance < 80%; OR: odds ratio; CI: confidence interval; AQ-Short: abridged version of the Autism Spectrum Quotient; ASRS: Adult ADHD Self-Report Scale; ADHD: attention deficit/hyperactivity disorder. Logistic regression was performed to estimate the OR and 95% CI for low attendance, using the score quartiles of the trait of developmental disability. The models were adjusted for age, gender, length of study, field of study, leisure-time physical activity, skipping breakfast, and living arrangement. ^a^In the first model, autistic traits and ADHD traits were entered simultaneously. ^b^In the second model, five autistic trait subcomponents and ADHD traits were entered simultaneously. The Hosmer–Lemeshow test showed a chi-square value of 8.39 and a *p*-value of 0.40 in the first model, and a chi-square value of 12.27 and a *p*-value of 0.14 in the second model.

Table 4-2 shows statistical associations between poor student performance (presenteeism) in university and scores on ADHD trait subcomponents. Univariate analyses revealed that participants in the highest Inattention trait score quartile and the highest Hyperactivity–Impulsivity trait score tertile were significantly more likely to report poor student performance: OR = 3.95 (95% CI: 1.92–8.12) and OR = 2.15 (95% CI: 1.33–3.47), respectively. In the third multivariate adjustment model (into which the autistic traits and two ADHD trait subcomponents had been entered simultaneously), participants in the highest Inattention trait score quartile were more likely to report poor student performance (OR = 2.88, 95% CI: 1.32–6.26). However, there were no statistically significant associations between the Hyperactivity–Impulsivity traits and poor student performance (OR = 1.54, 95% CI: 0.92–2.59). Furthermore, there were no statistically significant associations between ADHD trait subcomponents and low class attendance in either the univariate model or the multivariate models (Table 5-2).

**Table 4-2.** Association between Poor Student Performance (Presenteeism) in University and Scores on ADHD Trait Subcomponents (*n* = 721).

		Range of scores	No. of participants	No. of cases	Univariate		Multivariate^a^	
					**OR (95% CI)**	***p***	**OR (95% CI)**	***p***
**Autistic traits**								
AQ-Short	1^st^ Quartile (lowest)	40–66	197	44	1.00	-	1.00	-
	2^nd^ Quartile	67–70	182	43	1.08 (0.67–1.74)	0.765	1.04 (0.63–1.70)	0.884
	3^rd^ Quartile	71–75	192	70	2.00 (1.28–3.12)	0.002	1.93 (1.21–3.08)	0.006
	4^th^ Quartile (highest)	76–104	150	75	3.48 (2.19–5.53)	<0.001	2.85 (1.76–4.63)	<0.001
**ADHD trait subcomponents**								
Inattention	1^st^ Quartile (lowest)	0–3	110	28	1.00	-	1.00	-
	2^nd^ Quartile	4	222	56	0.99 (0.58–1.67)	0.964	0.99 (0.57–1.72)	0.969
	3^rd^ Quartile	5	342	121	1.60 (0.99–2.60)	0.055	1.38 (0.82–2.33)	0.224
	4^th^ Quartile (highest)	6–11	47	27	3.95 (1.92–8.12)	<0.001	2.88 (1.32–6.26)	0.008
Hyperactivity–impulsivity	1^st^ Tertile (lowest)	0–3	135	38	1.00	-	1.00	-
	2^nd^ Tertile	4	413	115	0.99 (0.64–1.52)	0.946	0.82 (0.51–1.31)	0.397
	3^rd^ Tertile (highest)	5-9	173	79	2.15 (1.33–3.47)	0.002	1.54 (0.92–2.59)	0.104

Poor performance: presenteeism scores ≤ 40; AQ-Short: abridged version of the Autism Spectrum Quotient; ASRS: Adult ADHD Self-Report Scale; ADHD: attention deficit/hyperactivity disorder. Logistic regression was performed to estimate the odds ratio (OR) and 95% confidence interval (CI) for poor performance, using the score quartiles of the developmental disability traits. Only the ADHD trait subcomponent “Hyperactivity–impulsivity” was analyzed in tertiles. The models were adjusted for age, gender, length of study, field of study, leisure-time physical activity, skipping breakfast, and living arrangement. ^a^In the third model, autistic traits, and ADHD trait subcomponents were entered simultaneously. The Hosmer–Lemeshow test showed a chi-square value of 7.28 and a *p*-value of 0.51 for poor performance in the third model.

**Table 5-2.** Association between Low Class Attendance in University and Scores on ADHD Trait Subcomponents (*n* = 721).

		Range of scores	No. of participants	No. of cases	Univariate		Multivariate^a^	
					**OR (95% CI)**	***p***	**OR (95% CI)**	***p***
**Autistic traits**								
AQ-Short	1^st^ Quartile (lowest)	40–66	197	34	1.00	-	1.00	-	
	2^nd^ Quartile	67–70	182	27	0.84 (0.48–1.45)	0.522	0.83 (0.47–1.47)	0.530	
	3^rd^ Quartile	71–75	192	33	1.00 (0.59–1.68)	0.985	0.91 (0.52–1.57)	0.722	
	4^th^ Quartile (highest)	76–104	150	28	1.10 (0.63–1.91)	0.735	0.93 (0.52–1.68)	0.814	
**ADHD trait subcomponents**									
Inattention	1^st^ Quartile (lowest)	0–3	110	15	1.00	-	1.00	-	
	2^nd^ Quartile	4	222	29	0.95 (0.49–1.86)	0.885	1.04 (0.52–2.08)	0.915	
	3^rd^ Quartile	5	342	66	1.51 (0.83–2.78)	0.180	1.71 (0.90–3.26)	0.105	
	4^th^ Quartile (highest)	6–11	47	12	2.17 (0.93–5.09)	0.075	2.23 (0.89–5.60)	0.088	
Hyperactivity–impulsivity									
	1^st^ Tertile (lowest)	0–3	135	20	1.00	-	1.00	-	
	2^nd^ Tertile	4	413	67	1.11 (0.65–1.92)	0.698	0.92 (0.52–1.64)	0.776	
	3^rd^ Tertile (highest)	5–9	173	35	1.46 (0.80–2.66)	0.220	1.11 (0.58–2.12)	0.750	

Low attendance: class attendance < 80%; AQ-Short: abridged version of the Autism Spectrum Quotient; ASRS: Adult ADHD Self-Report Scale; ADHD: attention deficit/hyperactivity disorder. Logistic regression was performed to estimate the odds ratio (OR) and 95% confidence interval (CI) for low attendance, using the score quartiles of the developmental disability traits. Only the ADHD trait subcomponent “Hyperactivity–impulsivity” was analyzed in tertiles. The models were adjusted for age, gender, length of study, field of study, leisure-time physical activity, skipping breakfast, and living arrangement. ^a^In the third model, autistic traits and ADHD trait subcomponents were entered simultaneously. The Hosmer–Lemeshow test showed a chi-square value of 6.91 and a *p*-value of 0.55 for low class attendance in the third model.

Table 4-3 shows statistical associations between poor student performance (presenteeism) in university and a combination of autistic trait scores and ADHD trait scores. Univariate analyses revealed that participants were significantly more likely to report poor student performance when either autistic traits or ADHD traits were “high” (OR = 2.78, 95% CI: 1.78–4.34; and OR = 2.14, 95% CI: 1.41–3.25, respectively), and that participants with both “high” autistic traits and “high” ADHD traits were more likely to report poor student performance than participants with either “high” autistic traits or “high” ADHD traits (OR = 4.31, 95% CI: 2.32–7.99). In the multivariate adjustment model, the results were essentially unchanged. Participants were significantly more likely to report poor student performance when they showed only “high” autistic traits, only “high” ADHD traits, and both “high” autistic traits and “high” ADHD traits (OR = 2.79, 95% CI: 1.78–4.39; OR = 2.20, 95% CI: 1.44–3.36; and OR = 3.78, 95% CI: 2.01–7.08, respectively). There were no statistically significant associations between “high” autistic traits and/or ADHD traits and low class attendance in either the univariate model or the multivariate model in the current study (Table 5-3).

**Table 4-3.** Association between Poor Student Performance (Presenteeism) in University and a Combination of Autistic Trait Scores and ADHD Trait Scores (*n* = 721).

**Autistic traits**		**ADHD traits**		No. of participants	No. of cases	Univariate		Multivariate	
AQ-Short	Range of scores	ASRS	Range of scores			**OR (95% CI)**	***p***	**OR (95% CI)**	***p***
Low	40–75	Low	0–9	444	106	1.00	-	1.00	-
High	76–104	Low	0–9	103	48	2.78 (1.78–4.34)	<0.001	2.79 (1.78–4.39)	<0.001
Low	40–75	High	10–20	127	51	2.14 (1.41–3.25)	<0.001	2.20 (1.44–3.36)	<0.001
High	76–104	High	10–20	47	27	4.31 (2.32–7.99)	<0.001	3.78 (2.01–7.08)	<0.001

Poor performance: presenteeism scores ≤ 40; the 4^th^ quartile (highest) of the developmental disability traits was defined as “high,” and the 1st quartile (lowest) to the 3rd quartile of these traits was defined as “low.” OR: odds ratio; CI: confidence interval; AQ-Short: abridged version of the Autism Spectrum Quotient; ASRS: Adult ADHD Self-Report Scale; ADHD: attention deficit/hyperactivity disorder. Logistic regression was performed to estimate the OR and 95% CI for poor performance, using the developmental disability trait score. The models were adjusted for age, gender, length of study, field of study, leisure-time physical activity, skipping breakfast, and living arrangement. The Hosmer–Lemeshow test in the multivariate model showed a chi-square value of 5.93 and a *p*-value of 0.66 in the cases of poor performance.

**Table 5-3.** Association between Low Class Attendance in University and a Combination of Autistic Trait Scores and ADHD Trait Scores (*n* = 721).

**Autistic traits**		**ADHD traits**		No. of participants	No. of cases	Univariate		Multivariate	
AQ-Short	Range of scores	ASRS	Range of scores			**OR (95% CI)**	***p***	**OR (95% CI)**	***p***
Low	40–75	Low	0–9	444	67	1.00	-	1.00	-
High	76–104	Low	0–9	103	20	1.36 (0.78–2.36)	0.281	1.48 (0.84–2.62)	0.180
Low	40–75	High	10–20	127	27	1.52 (0.92–2.50)	0.100	1.61 (0.97–2.69)	0.068
High	76–104	High	10–20	47	8	1.15 (0.52–2.58)	0.727	1.00 (0.43–2.31)	0.995

Low attendance: class attendance < 80%; the 4^th^ quartile (highest) of the developmental disability traits was defined as “high,” and the 1st quartile (lowest) to the 3rd quartile of these traits was defined as “low.” OR: odds ratio; CI: confidence interval; AQ-Short: abridged version of the Autism Spectrum Quotient; ASRS: Adult ADHD Self-Report Scale; ADHD: attention deficit/hyperactivity disorder. Logistic regression was performed to estimate the OR and 95% CI for low class attendance, using the developmental disability trait score. The models were adjusted for age, gender, length of study, field of study, leisure-time physical activity, skipping breakfast, and living arrangement. The Hosmer–Lemeshow test in the multivariate model showed a chi-square value of 7.21 and a *p*-value of 0.51 in the cases of low class attendance.

## Discussion

The simultaneous analysis of autistic traits and ADHD traits in a multivariate adjusted model revealed that students with high levels of autistic and ADHD traits were significantly more likely to report poor student performance (i.e., sickness presenteeism). The simultaneous analysis of autistic trait subcomponents and ADHD traits revealed that poor student performance (sickness presenteeism) was statistically associated with Routine traits and Social skills traits, and weakly associated with Switching traits. However, there were no demonstrable associations between autistic traits or ADHD traits and low class attendance risk. These findings suggest that high levels of autistic and ADHD traits probably affect student performance, but are less influential on class attendance.

The current study revealed statistically significant associations between autistic or ADHD traits and poor student performance. Although, to the best of our knowledge, no previous reports demonstrate an association between autistic traits and poor student performance, some research has documented an association between ASD and both academic achievement and driving. There are some parallels with our current study of students’ academic achievement, as driving can also be viewed as a “performance” task, of sorts.

Previous reports have shown that the academic achievement of students with ASD is often lower than would be expected based on their IQ levels alone ^(5), (33)^, suggesting that students with ASD are underperforming relative to their ability level. Students with ASD are also reported to experience high levels of behavioral and emotional difficulties at school, such as anxiety and depression ^(5)^. Individuals with high autistic traits with or without ASD diagnosis are at a high risk of associated psychiatric disorders, particularly depression and anxiety ^(34), (35), (36)^. In terms of driving, individuals with ASD have been shown to underperform in their driving ability and are involved in more traffic accidents than drivers without ASD, although individuals with ASD outperform on aspects related to rule-following ^(37), (38), (39)^. These findings when viewed in the context of our own results suggest that individuals with ASD/high levels of autistic traits probably struggle to concentrate and to calm their emotions and behaviors during class. From another perspective, one of the characteristics of ASD is the presence of restricted, repetitive behaviors, interests, or activities ^(2)^. Therefore, it is difficult for students with ASD/high levels of autistic traits to follow university rules set by others, resulting in poor student performance. The current study also suggests that difficulties due to high levels of autistic traits probably manifest in poor student performance, which subsequently leads to poor academic achievement, as students are unable to demonstrate their true abilities. Poor academic achievement in the current research was affected not only by ability but also by poor student performance (i.e., sickness presenteeism), suggesting that high autistic traits or ASD might be generally more prevalent among less-educated people ^(6), (7)^.

Our results, demonstrating an association between ADHD traits and poor student performance, are congruent with other research, including a previous study of employees that showed that ADHD is associated with a 4–5% reduction in work performance as measured by the WHO-HPQ ^(11)^. In the present study, we used the WHO-HPQ to assess student performance. Previous studies on students have revealed that the ADHD traits of hyperactivity, impulsivity, and inattention can affect school performance ^(40), (41)^. Several studies have also reported an association between ADHD traits and student academic achievement, for example, individuals who exhibit symptoms of inattention, hyperactivity, and impulsivity (with or without ADHD diagnosis) also suffer poor academic and educational outcomes ^(9), (42)^. One previous study even found that the association between ADHD and academic underachievement persisted after adjusting for IQ ^(43)^. This suggests that academic achievement is affected by aspects of student performance, such as presenteeism, rather than being a direct reflection of academic ability. Lower than expected academic achievement in students who have sufficient ability may indicate mental stress and a tendency to depression. Evidence shows that individuals with ADHD or probable ADHD are at a high risk of associated psychiatric disorders, such as depression and social anxiety ^(44), (45), (46)^. Social anxiety also has a substantial effect on failure to complete school, and increases the risk of examination failure ^(47)^.

Analyses of the five autistic trait subcomponents, ADHD, and poor student performance showed that scores on all autistic trait subcomponents except two (Numbers/Patterns and Imagination) were associated with poor student performance. Of the five subcomponents, there was a strong effect size for Routine traits, but weak effect sizes for Social skills and Switching traits. We assumed that student performance would be strongly associated with the Social skills trait, resulting in lower academic achievement, based on previous reports that have shown that improved social skills lead to improved academic achievement ^(33), (48), (49)^. Interpersonal relationships are important for performance in practical classes at university. However, students often study alone, so social skills traits may have little effect on performance. Individuals with ASD tend to perform lower than their typically developing peers on reading comprehension ^(49), (50)^. Taken together with our results, these findings suggest that individuals with high levels of autistic traits may prefer routine to avoid reading comprehension, and as a result, do not improve their reading skills and consequently exhibit poor student performance. Our previous study uniquely showed that workers of higher SES scored higher on the Numbers/Patterns trait, although workers of lower SES had significantly higher autistic trait scores than their respective counterparts ^(7)^. Baron-Cohen et al. ^(22), (51)^ found that mathematical talent is linked to autism. The autistic traits indicated by Baron-Cohen et al. resemble the Numbers/Patterns trait. These findings suggest that the Numbers/Patterns trait may not be associated with poor student performance because it has positive aspects. We found that the Imagination trait was not independently associated with poor student performance. Individuals who score highly on the Imagination trait find it difficult to understand another person’s point of view. However, individuals need other people (i.e., interpersonal relationships). This suggests that the lack of association between Imagination trait scores and poor student performance reflects the fact that students often study alone.

Analyses of the two ADHD trait subcomponents and poor student performance showed that scores on the Inattention trait were associated with poor student performance. However, we found that the Hyperactivity–Impulsivity trait was not independently associated with poor student performance. This seems to support previous study findings that hyperactivity/impulsivity symptoms present in childhood decline with age, whereas inattention symptoms persist into adulthood, and most adults with ADHD present with prominent symptoms of inattention ^(52), (53)^.

The analysis of a combination of autistic trait scores and ADHD trait scores and poor student performance showed that participants with either “high” autistic traits or “high” ADHD trait scores were significantly more likely to report poor student performance, and that participants with both “high” autistic traits and ADHD trait scores were more likely to report poor student performance than those with only “high” scores on one trait type. DSM-5 no longer prohibits the co-diagnosis of ASD and ADHD, and recent studies indicate that the co-occurrence of ADHD and autistic symptoms is common ^(54), (55)^. Our results are consistent with previous reports that individuals with both ASD and ADHD have higher rates of other comorbid symptoms than individuals with either ASD or ADHD alone ^(56), (57)^.

In the current study, we found no statistically significant association between low class attendance risk and autistic traits, autistic trait subcomponents, or ADHD traits. A previous study on workers revealed that ADHD was associated with reduced work performance and the relative odds of sickness absence were 2.1 ^(11)^, which contrasts with our findings whereby high levels of autistic or ADHD traits affect student performance, but do not affect class attendance, suggesting that poor student performance owing to these traits does not necessarily lead to low class attendance. Our outcome measure of class attendance included not only absences from class owing to illness but also cases where students skipped class. In previous studies on children or students aged 5–17 years, individuals with ASD or ADHD were more likely to have shown chronic school absenteeism, school dropout, or school refusal behavior than individuals without these conditions ^(58), (59), (60)^. These previous studies are inconsistent with our results. This may be because our participants were university students, and therefore highly motivated to study compared with the samples of children or students aged 5–17 years in the previous studies.

About half of our participants had autistic trait scores above the cutoff and 18% of participants had ADHD trait scores above the cutoff. These proportions are high compared with the prevalence of both previously reported ASD (0.98% to 2.2%) ^(6), (61)^ and DSM-5 ADHD (3.55%) ^(62)^. When limited to university students, the prevalence of ASD without co-occurring intellectual disability is 1–2% ^(63)^, and approximately 2–8% of students show clinically significant levels of ADHD ^(64)^. The Japan Student Services Organization reported that only 0.2% of college students in Japan have developmental disabilities, 56.7% have ASD, 25.2% have ADHD, and 14.7% have two or more concurrent developmental disabilities ^(65)^. These estimates are based only on students with a certificate of disability or those whose disability was revealed by a medical examination, which may indicate that the number of students with developmental disabilities has been underestimated. There are several possible reasons for the high proportion of students with developmental disabilities in this study compared with previous reports. First, some individuals have high levels of autistic traits/ADHD traits, but do not have an ASD/ADHD diagnosis. Second, many participants in this study with high levels of autistic traits and/or ADHD traits may have participated because they were interested in exploring their own high levels of autistic/ADHD traits. Additionally, participants were university students and so belonged to a rather high SES group. More individuals with high autistic traits and/or ADHD traits may have been attracted to the study because of social class bias. Owing to the presence of this bias, high SES is a reported sociodemographic risk factor for ASD ^(66), (67)^. For instance, children with autism whose mothers are more highly educated may have had more opportunity to participate in early interventions than children whose mothers have had fewer years of education ^(66), (67)^.

### Limitations

There were a few potential limitations of the current study that are worth considering. First, our questionnaire may be less appropriate for participants with a low IQ, as it required a certain degree of reading comprehension skills. The participants in our study, therefore, presumably comprised those with typical intelligence levels. Second, as we used an online questionnaire, the sample may have been biased toward individuals who owned a computer and were more familiar with internet use. Third, the current study utilized self-reporting measures, which may exhibit an inherent degree of recall error and bias (particularly regarding class attendance), although this methodology is by no means uncommon in psychological research ^(68), (69)^.

### Implications

The implications of our study are that the students with high levels of autistic and ADHD traits were significantly more likely to report poor student performance, although there was no association between autistic traits or ADHD traits and low class attendance risk. Attendance and absence are easy to distinguish objectively, but poor student performance is harder to determine. It is important to notice poor student performance early and creating a suitable learning environment will improve their performance. It helps students attain an academic achievement that better reflects their ability.

### Conclusion

Overall, the current study suggests that poor student performance as measured by sickness presenteeism was significantly associated with high levels of both autistic traits and ADHD traits. Low class attendance risk was not statistically associated with either autistic or ADHD traits, however. The findings revealed that while high levels of autistic and ADHD traits affect student performance, class attendance rates are less affected, at least in Japan.

## Article Information

### Conflicts of Interest

None

### Sources of Funding

This work was supported by the Japan Society for the Promotion of Science (JSPS) KAKENHI [grant number JP16K09105 (Chief: Dr. Tomoko Suzuki)] and IUHW Research Grants (Chief: Dr. Tomoko Suzuki), Japan.

### Acknowledgement

The authors would like to acknowledge Dr. Baron-Cohen’s permission to create our Japanese version of the AQ-Short questionnaire. We thank Dr. Derek R. Smith for his helpful advice. We would also like to express our sincere gratitude to all participants for their involvement in our research. We also thank those who cooperated with us during data collection and entry.

### Author Contributions

TS conceived the study, designed the protocol, and enrolled participants, and was responsible for the statistical analysis and drafting of the manuscript. SI and TS were responsible for the organization of the entire cohort and participated in data collection. KW was the advisor for the whole study and completed the manuscript. BM, NN, and SY gave valuable advice and revised the manuscript for important intellectual content. All the authors were involved in the data interpretation and contributed to preparing the manuscript.

### Approval by Institutional Review Board (IRB)

Ethical approval was obtained from the International University of Health and Welfare Ethics Committee (No. 16-Ig-62) and the Kitasato University School of Medicine Ethics Committee (No. B16-160).

## Supplement

Supplementary Material 1-5.
