# Supplementary material for: Autistic and Attention Deficit/Hyperactivity Disorder Traits Are Associated with Suboptimal Performance among Japanese University Students
Source: JMA J. 2020 Jul 7;3(3):216–31. doi: 10.31662/jmaj.2020-0001 (PMC7590390; doi:10.31662/jmaj.2020-0001)
Supplement: Supplementary file 1 — Supplementary Material 1-5. [file 2433-3298-3-3-0216-s001.pdf]

Supplementary Table 1. Participant scores on autistic traits and ADHD traits

	n	Autistic traits (AQ Short)			ADHD traits (ASRS)			Autistic traits (AQ-Short) & ADHD traits (ASRS)				
			<70 [a]	≥ 70[a]		<11[a]	≥ 11[a]	AQS	<70[a]	≥ 70[a]	<70[a]	≥ 70[a]
								ADHD	<11[a]	<11[a]	≥ 11[a]	≥ 11[a]
Total	721	70.5±8	327 (45.4)	394 (54.6)	8.5±2.5	588 (81.6)	133 (18.4)		275 (38.1)	313 (43.4)	52 (7.2)	81 (11.2)
Male	218	70.8±7.1	90 (41.3)	128 (58.7)	8.4±2.7	173 (79.4)	45 (20.6)		76 (34.9)	97 (44.5)	14 (6.4)	31 (14.2)
Female	503	70.4±8.4	237 (47.1)	266 (52.9)	8.6±2.5	415 (82.5)	88 (17.5)		199 (39.6)	216 (42.9)	38 (7.6)	50 (9.9)

AQ-Short: abridged version of the Autism Spectrum Quotient; ASRS: Adult ADHD Self-Report Scale; ADHD: attention deficit/hyperactivity disorder. Continuous variables are expressed as mean ± standard deviation. Categorical variables are expressed as numbers (percentages); [a]Cut-off value from reference [21, 26].

Supplementary Table 2. Multivariate analysis details of Table 4-1. Association between poor student performance (presenteeism) in university and scores on autistic traits, autistic trait subcomponents, and ADHD traits (*n* = 721)

		Range of Scores	No. of participants	No. of poor performance	B coefficient	SE	Wald	df	**Multivariate**	
									**OR (95% CI)**	***p***
**(First model)**										
**Autistic traits**										
AQ Short	1st Quartile (lowest)	40-66	197	44					1.00	-
	2nd Quartile	67-70	182	43	0.03	0.25	0.01	1	1.03 (0.63-1.68)	0.915
	3rd Quartile	71-75	192	70	0.65	0.24	7.63	1	1.92 (1.21-3.04)	0.006
	4th Quartile (highest)	76-104	150	75	1.12	0.25	21.06	1	3.07 (1.90-4.96)	<0.001
**ADHD traits**										
ASRS	1st Quartile (lowest)	0-7	174	43					1.00	-
	2nd Quartile	8-8	141	36	0.02	0.27	0.00	1	1.02 (0.60-1.73)	0.952
	3rd Quartile	9-9	232	75	0.20	0.24	0.69	1	1.22 (0.77-1.93)	0.408
	4th Quartile (highest)	10-20	174	78	0.76	0.24	9.67	1	2.13 (1.32-3.42)	0.002
**(Second model)**										
**Autistic Traits Subcomponents**										
Numbers/patterns	1st Quartile (lowest)	5-9	192	75					1.00	-
	2nd Quartile	10-11	192	61	-0.10	0.24	0.18	1	0.90 (0.56-1.46)	0.675
	3rd Quartile	12-13	166	44	-0.28	0.26	1.14	1	0.76 (0.45-1.26)	0.285
	4th Quartile (highest)	14-20	171	52	0.01	0.27	0.00	1	1.01 (0.60-1.70)	0.968
Imagination	1st Quartile (lowest)	8-18	236	60					1.00	-
	2nd Quartile	19-20	198	57	0.15	0.24	0.41	1	1.16 (0.73-1.84)	0.523

	3rd Quartile	21-22	159	51	0.11	0.25	0.18	1	1.11 (0.68-1.81)	0.670
	4th Quartile (highest)	23-32	128	64	0.41	0.28	2.27	1	1.51 (0.88-2.59)	0.132
Routine	1st Quartile (lowest)	4-9	169	31					1.00	-
	2nd Quartile	10-10	173	57	0.71	0.27	6.73	1	2.03 (1.19-3.48)	0.010
	3rd Quartile	11-11	164	43	0.31	0.29	1.17	1	1.37 (0.78-2.40)	0.280
	4th Quartile (highest)	12-16	215	101	0.87	0.28	9.74	1	2.39 (1.38-4.13)	0.002
Social skills	1st Quartile (lowest)	7-15	186	48					1.00	-
	2nd Quartile	16-18	225	53	-0.25	0.25	0.99	1	0.78 (0.48-1.27)	0.321
	3rd Quartile	19-21	175	59	0.17	0.26	0.43	1	1.18 (0.71-1.97)	0.515
	4th Quartile (highest)	22-28	135	72	0.59	0.29	4.20	1	1.81 (1.03-3.18)	0.040
Switching	1st Quartile (lowest)	6-9	214	51					1.00	-
	2nd Quartile	10-11	274	77	0.03	0.23	0.02	1	1.03 (0.66-1.62)	0.893
	3rd Quartile	12-12	111	37	-0.01	0.30	0.00	1	0.99 (0.55-1.78)	0.969
	4th Quartile (highest)	13-16	122	67	0.57	0.29	3.84	1	1.78 (1.00-3.15)	0.050
**ADHD traits**										
ASRS	1st Quartile (lowest)	0-7	174	43					1.00	-
	2nd Quartile	8-8	141	36	0.21	0.28	0.55	1	1.23 (0.71-2.15)	0.459
	3rd Quartile	9-9	232	75	0.30	0.25	1.50	1	1.35 (0.83-2.20)	0.220
	4th Quartile (highest)	10-20	174	78	0.80	0.26	9.88	1	2.24 (1.35-3.69)	0.002

Poor performance: presenteeism scores ≤40; B: beta; SE: standard error; df: degree of freedom; OR: odds ratio; CI: confidence interval; AQ-Short: abridged version of the Autism Spectrum Quotient; ASRS: Adult ADHD Self-Report Scale; ADHD: attention deficit/hyperactivity disorder. Logistic regression was performed to estimate the odds ratio (OR) and 95% confidence interval (CI) for poor performance, using the score quartiles of the trait of developmental disability. The models were adjusted for age, gender, length of

study, field of study, leisure-time physical activity, skipping breakfast, and living arrangement. In the first model, autistic traits and ADHD traits were entered simultaneously. In the second model, five autistic trait subcomponents and ADHD traits were entered simultaneously. The Hosmer–Lemeshow test showed a chi-square value of 5.44, and a *p* value of 0.71 in the first model, and a chi-square value of 7.54, and a *p* value of 0.48 in the second model.

Supplementary Table 3. Stepwise multivariate logistic regression of the association between poor student performance (presenteeism) in university and scores on autistic trait subcomponents, and ADHD traits (n = 721)

		Range of Scores	No. of participants	No. of poor performance	B coefficient	SE	Wald	df	**Multivariate**	
									**OR (95% CI)**	***p***
**(Second model)**										
**Autistic Traits Subcomponents**										
Numbers/patterns	1st Quartile (lowest)	5-9	192	75						
	2nd Quartile	10-11	192	61						
	3rd Quartile	12-13	166	44						
	4th Quartile (highest)	14-20	171	52						
Imagination	1st Quartile (lowest)	8-18	236	60						
	2nd Quartile	19-20	198	57						
	3rd Quartile	21-22	159	51						
	4th Quartile (highest)	23-32	128	64						
Routine	1st Quartile (lowest)	4-9	169	31					1.00	-
	2nd Quartile	10-10	173	57	0.75	0.27	7.80	1	2.11 (1.25-3.56)	0.005
	3rd Quartile	11-11	164	43	0.35	0.28	1.57	1	1.43 (0.82-2.48)	0.210
	4th Quartile (highest)	12-16	215	101	0.88	0.27	10.49	1	2.41 (1.42-4.11)	0.001
Social skills	1st Quartile (lowest)	7-15	186	48					1.00	-
	2nd Quartile	16-18	225	53	-0.21	0.24	0.78	1	0.81 (0.50-1.30)	0.377
	3rd Quartile	19-21	175	59	0.19	0.25	0.55	1	1.20 (0.74-1.97)	0.461
	4th Quartile (highest)	22-28	135	72	0.72	0.28	6.80	1	2.05 (1.20-3.51)	0.009
Switching	1st Quartile (lowest)	6-9	214	51					1.00	-

	2nd Quartile	10-11	274	77	0.03	0.22	0.02	1	1.03 (0.67-1.60)	0.881
	3rd Quartile	12-12	111	37	0.06	0.28	0.04	1	1.06 (0.61-1.84)	0.843
	4th Quartile (highest)	13-16	122	67	0.70	0.27	6.51	1	2.01 (1.18-3.45)	0.011
**ADHD traits**										
ASRS	1st Quartile (lowest)	0-7	174	43					1.00	-
	2nd Quartile	8-8	141	36	0.25	0.28	0.80	1	1.28 (0.75-2.20)	0.371
	3rd Quartile	9-9	232	75	0.33	0.24	1.87	1	1.39 (0.87-2.22)	0.172
	4th Quartile (highest)	10-20	174	78	0.83	0.25	11.17	1	2.30 (1.41-3.73)	0.001

Poor performance: presenteeism scores ≤40; B: beta; SE: standard error; df: degree of freedom; OR: odds ratio; CI: confidence interval; ASRS: Adult ADHD Self-Report Scale; ADHD: attention deficit/hyperactivity disorder. Stepwise multivariate logistic regression was performed to estimate the odds ratio (OR) and 95% confidence interval (CI) for poor performance, using the score quartiles of the developmental disability traits. The models were adjusted for age, gender, length of study, field of study, leisure-time physical activity, skipping breakfast, and living arrangement. In the second model, five autistic trait subcomponents and ADHD traits were entered simultaneously. The Hosmer-Lemeshow test showed a chi-square value of 6.09, and a *p* value of 0.64.

Supplementary Table 4. Multivariate analysis details of Table 5-1. Association between low class attendance in university and scores on autistic traits, autistic traits subcomponents, and ADHD traits (n = 721)

		Range of Scores	No. of participants	No. of low class attendance	B coefficient	SE	Wald	df	Multivariate[a]	
									**OR (95%CI)**	***p***
**(First model)**										
**Autistic traits**										
AQ Short	1st Quartile (lowest)	40-66	197	34					1.00	-
	2nd Quartile	67-70	182	27	-0.18	0.29	0.38	1	0.84 (0.47-1.48)	0.536
	3rd Quartile	71-75	192	33	-0.08	0.28	0.09	1	0.92 (0.53-1.60)	0.768
	4th Quartile (highest)	76-104	150	28	0.00	0.30	0.00	1	1.00 (0.56-1.79)	0.996
**ADHD traits**										
ASRS	1st Quartile (lowest)	0-7	174	26					1.00	-
	2nd Quartile	8-8	141	16	-0.27	0.35	0.60	1	0.76 (0.39-1.51)	0.439
	3rd Quartile	9-9	232	45	0.36	0.28	1.59	1	1.43 (0.82-2.48)	0.207
	4th Quartile (highest)	10-20	174	35	0.37	0.30	1.58	1	1.45 (0.81-2.59)	0.209
**(Second model)**										
**Autistic Traits Subcomponents**										
Numbers/patterns	1st Quartile (lowest)	5-9	192	32					1.00	-
	2nd Quartile	10-11	192	29	0.05	0.31	0.02	1	1.05 (0.57-1.91)	0.883
	3rd Quartile	12-13	166	33	0.52	0.31	2.81	1	1.68 (0.92-3.07)	0.094
	4th Quartile (highest)	14-20	171	28	0.08	0.33	0.06	1	1.09 (0.57-2.08)	0.802
Imagination	1st Quartile (lowest)	8-18	236	41					1.00	-

	2nd Quartile	19-20	198	34	-0.09	0.28	0.09	1	0.92 (0.53-1.58)	0.759
	3rd Quartile	21-22	159	25	-0.30	0.30	0.97	1	0.74 (0.41-1.35)	0.325
	4th Quartile (highest)	23-32	128	22	-0.15	0.35	0.19	1	0.86 (0.44-1.69)	0.661
Routine	1st Quartile (lowest)	4-9	169	26					1.00	-
	2nd Quartile	10-10	173	27	0.03	0.32	0.01	1	1.03 (0.55-1.93)	0.935
	3rd Quartile	11-11	164	30	0.30	0.32	0.88	1	1.35 (0.72-2.54)	0.347
	4th Quartile (highest)	12-16	215	39	0.16	0.33	0.23	1	1.17 (0.61-2.24)	0.635
Social skills	1st Quartile (lowest)	7-15	186	37					1.00	-
	2nd Quartile	16-18	225	29	-0.61	0.29	4.37	1	0.54 (0.31-0.96)	0.036
	3rd Quartile	19-21	175	27	-0.45	0.31	2.10	1	0.64 (0.35-1.17)	0.147
	4th Quartile (highest)	22-28	135	29	-0.10	0.34	0.08	1	0.91 (0.47-1.78)	0.780
Switching	1st Quartile (lowest)	6-9	214	36					1.00	-
	2nd Quartile	10-11	274	43	-0.12	0.27	0.20	1	0.89 (0.52-1.51)	0.659
	3rd Quartile	12-12	111	13	-0.29	0.39	0.55	1	0.75 (0.35-1.61)	0.459
	4th Quartile (highest)	13-16	122	30	0.40	0.34	1.36	1	1.49 (0.76-2.92)	0.244
**ADHD traits**										
ASRS	1st Quartile (lowest)	0-7	174	26					1.00	-
	2nd Quartile	8-8	141	16	-0.20	0.36	0.30	1	0.82 (0.41-1.67)	0.586
	3rd Quartile	9-9	232	45	0.43	0.29	2.17	1	1.54 (0.87-2.73)	0.140
	4th Quartile (highest)	10-20	174	35	0.37	0.31	1.46	1	1.45 (0.79-2.64)	0.227

Low attendance: class attendance< 80%; OR: odds ratio; CI: confidence interval; B: beta; SE: standard error; df: degree of freedom; AQ-Short: An Abridged Version of the Autism-Spectrum Quotient; ASRS: Adult ADHD Self-Report Scale; Logistic regression was performed to estimate the odds ratio (OR) and 95% confidence interval (CI) for low attendance, using the scores quartiles of the trait of developmental

disability. The models were adjusted for age, gender, length of study, field of study, leisure-time physical activity, skipping breakfast, and living arrangement. In the first model, autistic traits and ADHD traits were entered simultaneously. In the second model, five autistic trait subcomponents and ADHD traits were entered simultaneously. The Hosmer–Lemeshow test showed a chi-square value of 8.39, and a *p* value of 0.40 in the first model, and a chi-square value of 12.27, and a *p* value of 0.14 in the second model.

Supplementary Table 5. Stepwise multivariate logistic regression of the association between low class attendance in university and scores on autistic traits subcomponents, and ADHD traits. (n = 721)

		Range of Scores	No. of participants	No. of low class attendance	B coefficient	SE	Wald	df	Multivariate[a]	
									**OR (95%CI)**	***p***
**(Second model)**										
**Autistic Traits Subcomponents**										
Numbers/patterns	1st Quartile (lowest)	5-9	192	32						
	2nd Quartile	10-11	192	29						
	3rd Quartile	12-13	166	33						
	4th Quartile (highest)	14-20	171	28						
Imagination	1st Quartile (lowest)	8-18	236	41						
	2nd Quartile	19-20	198	34						
	3rd Quartile	21-22	159	25						
	4th Quartile (highest)	23-32	128	22						
Routine	1st Quartile (lowest)	4-9	169	26						
	2nd Quartile	10-10	173	27						
	3rd Quartile	11-11	164	30						
	4th Quartile (highest)	12-16	215	39						
Social skills	1st Quartile (lowest)	7-15	186	37					1.00	-
	2nd Quartile	16-18	225	29	-0.58	0.28	4.34	1	0.56 (0.33-0.97)	0.037
	3rd Quartile	19-21	175	27	-0.37	0.28	1.71	1	0.69 (0.40-1.20)	0.190
	4th Quartile (highest)	22-28	135	29	0.04	0.29	0.02	1	1.04 (0.60-1.83)	0.881

Switching	1st Quartile (lowest)	6-9	214	36
	2nd Quartile	10-11	274	43
	3rd Quartile	12-12	111	13
	4th Quartile (highest)	13-16	122	30
**ADHD traits**				
ASRS	1st Quartile (lowest)	0-7	174	26
	2nd Quartile	8-8	141	16
	3rd Quartile	9-9	232	45
	4th Quartile (highest)	10-20	174	35

Low attendance: class attendance< 80%; OR: odds ratio; CI: confidence interval; B: beta; SE: standard error; df: degree of freedom; ASRS: Adult ADHD Self-Report Scale; Stepwise multivariate logistic regression was performed to estimate the odds ratio (OR) and 95% confidence interval (CI) for low attendance, using the scores quartiles of the trait of developmental disability. The models were adjusted for age, gender, length of study, field of study, leisure-time physical activity, skipping breakfast, and living arrangement. In the second model, five autistic trait subcomponents and ADHD traits were entered simultaneously. The Hosmer-Lemeshow test showed a chi-square value of 5.18, and a p value of 0.64.
